# Explaining Regeneration: Cells and Limbs as Complex Living Systems, Learning From History

**DOI:** 10.3389/fcell.2021.734315

**Published:** 2021-08-31

**Authors:** Kate MacCord, Jane Maienschein

**Affiliations:** ^1^School of Life Sciences, Arizona State University, Tempe, AZ, United States; ^2^Eugene Bell Center for Regenerative Biology and Tissue Engineering, Marine Biological Laboratory, Woods Hole, MA, United States

**Keywords:** regeneration, complex living systems, Morgan, generalizability, reductionism, model organisms, blastema

## Abstract

Regeneration has been investigated since Aristotle, giving rise to many ways of explaining what this process is and how it works. Current research focuses on gene expression and cell signaling of regeneration within individual model organisms. We tend to look to model organisms on the reasoning that because of evolution, information gained from other species must in some respect be generalizable. However, for all that we have uncovered about how regeneration works within individual organisms, we have yet to translate what we have gleaned into achieving the goal of regenerative medicine: to harness and enhance our own regenerative abilities. Turning to history may provide a crucial perspective in advancing us toward this goal. History gives perspective, allowing us to reflect on how our predecessors did their work and what assumptions they made, thus also revealing limitations. History, then, may show us how we can move from our current reductionist thinking focused on particular selected model organisms toward generalizations about this crucial process that operates across complex living systems and move closer to repairing our own damaged bodies.

## Introduction

Regeneration is a long-recognized phenomenon, dating back to Aristotle. Every species maintains some capacity to regenerate, though that capacity varies drastically. Although we have appreciated that organisms have the ability to repair and replace lost and damaged parts for over 2000 years, conceptualization of what regeneration is, how it works, and how best to explain it remain under construction. Each study of regeneration from cells to limbs today typically involves looking at a single species and examining molecular and genetic activation, or stem cells’ responses to environmental stimuli that damage an organism. Yet collectively, the research community increasingly conducts studies across a wide diversity of organisms, as witnessed in this special issue.

When compared to Hydra or axolotls, our own ability to regenerate is particularly poor. Yet we seek to do better—this is the goal of regenerative medicine, to harness and enhance our regenerative abilities for biomedical interventions. We look at how regeneration works in other species on the reasoning that because of evolution, information gained from other species must in some respect be generalizable. Since Aristotle, understanding of regeneration has certainly progressed, but we have yet to synthesize understanding of this process across species. Simply put, how can we explain regeneration in a way that is applicable beyond the individual species in each laboratory to the diversity of species on our planet and to our own damaged bodies?

In other words, we look toward an understanding of regeneration that is generalizable, such that knowledge of the process(es) of regeneration can be abstracted and applied from the multitude of studies on various parts of individuals to whole organisms and broadly across species. The attempt at generalizability will likely require more mathematical modeling and much more conscious coordination across laboratories studying different organisms. As historians and philosophers of science taking a broad view, we see efforts at generalization as tremendously valuable and also see indications that it is time to work in that direction. This effort will require embracing new ways of thinking and looking to history may help. History gives perspective, allowing us to reflect on how our predecessors did their work and on what assumptions they made, thus also revealing limitations. By exploring the history of gaining knowledge of nature, we move toward acquiring a deeper understanding of the nature of knowledge and in so doing, we can use the work of the past to shape the endeavors of the future.

### Regeneration Through History

What can history show about understanding regeneration? How have people investigated and explained regeneration, and what were their limitations and assumptions? Throughout the following sections, we quickstep through the long and storied history of regeneration research, synthesizing guiding principles, main ideas, and limitations of three periods.

Historical study of regeneration tends to start with Greek mythology such as stories of Prometheus, bound to a rock and destined to have his liver plucked out by an eagle each day only to have it regenerate each night, or Hercules’ battle against the nine-headed Hydra capable of quickly regenerating its lost heads. Despite some observations by that keen naturalist Aristotle, what we consider scientific awareness of regeneration began in the 18th century, when investigators began to recognize and record regeneration in earnest. A small battalion of experimenters, inspired by the era of exploration in which they lived, captured Hydra, worms, and other creatures and chopped them into pieces to observe what would happen and consider what it meant. Their driving questions centered around definitions of life: could these disembodied bits continue to “live,” and what did this say about whether life requires a vital force and whether organization of the whole has the capacity to direct its recovery? (Maienschein and MacCord, 2022).

These figures of the Enlightenment exhibited great curiosity and eagerness to discover what is inside organisms and what makes them whole. They also increasingly embraced the idea called materialism that living organisms as well as inanimate objects all consist of matter that is constantly in motion, and experimental approaches. They saw parts of organisms, but not anything called cells. And they focused on discovering and observing, while offering less in the way of explanation. For that, we move to the end of the 19th and into the 20th century.

### Toward Generalizability: Regeneration in Complex Living Systems

Beginning in the late 19th century, investigators who turned toward regeneration sought to understand it in ways that we would recognize, through materialistic, experimentally based explanations. Scientists like Thomas Hunt Morgan, Jacques Loeb, and Charles Manning Child understood organisms as made up of cells and took a systems-based approach in order to form generalizable explanations of how regeneration works. However, their generalizable explanations were too abstract and lacking in fine details to be tractable for use in controlling regeneration.

Morgan (1901) published *Regeneration*, a summary of previous studies and his own work on a diversity of organisms. There Morgan (1901) emphasized that, “the forming organism is of such a kind that we can better understand its action when we consider it as a whole and not simply as the sum of a vast number of smaller elements.” (p. 278) Organisms consist of cells, but it is the whole that matters for regeneration. Even though best known for his study of *Drosophila* genetics, Morgan resisted reductionistic tendencies to take the organism apart, to over-emphasize genes, and to lose track of the interacting whole. This emphasis also characterized the work of Morgan’s contemporaries Jacques Loeb and Charles Manning Child, each of whom saw regeneration as a valuable way to understand living systems. It is worth recalling what Morgan, Loeb, and Child were thinking at the beginning of the 20th century, why, and what we learn from this history.

These three men overlapped in many ways, including the questions they asked, organisms they studied, where they worked, and how they carried out their experiments. They all looked for explanations of regeneration in material terms. They all thought in terms of the whole organism as a living system, including its individuality and organization. They all demanded that explanations must be grounded in experimentally based evidence and avoided philosophical speculation far beyond their data. Yet they also had quite different accounts of regeneration.

While presenting his observations in his 1901 book and dozens of articles, Morgan noted that he had not reached an overarching theory to explain regeneration. He saw two different modes of regeneration. “Morphallaxis” occurs when the organism somehow causes existing material to reshape into the missing part, and “epimorphosis” involves production of new material. He saw these as descriptive terms, representing two different ways organisms can respond to injury. Yet he also suggested the more theoretical “tensions” within an organized self-regulating organism. Tensions hold the parts together in the right relationship, not allowing them to become too close nor to drift apart. Injury can disrupt the system’s balance, Morgan felt, by pulling the tensions out of order. Regeneration involves restoring that order. Morgan (1901) could not directly observe these tensions, so he made clear that he offered them as a working hypothesis, to be tested and refined.

Morgan carried out his work at Bryn Mawr College, then at Columbia University, and during summers at the Marine Biological Laboratory (MBL) in Woods Hole, Massachusetts, beginning in 1890. Loeb also began spending summers at the MBL in the early 1890s. There he took up questions about regeneration, with an even more ardent commitment to finding materialistic explanations. Like Morgan, Loeb also studied a diversity of different organisms, though he soon focused his regeneration studies on plants.

Where Morgan offered his working tensions hypothesis, Loeb focused on tropisms. A tropism is the movement of parts or the whole organism in response to an external stimulus. Unlike Morgan with his working hypothesis, Loeb offered his theory as the right one and sought evidence in its favor. In a 1907 article, Loeb acknowledged his interest in “controlling life.” (Pauly, 1987) “There may be a difference of opinion as to whether or not it will ever be possible to produce living matter from inanimate,” Loeb (1907) wrote, but “we cannot well hope to succeed in making living matter artificially unless we have a clear conception of what living matter is.” (p. 425) Biological investigation should ask what is living matter and how does it work in organized living individual organisms. It should also seek explanations in terms of quantitative studies and rigorous mathematical formulas and laws.

Loeb’s favorite research subject became *Bryophyllum calycinum*, called the life plant, that he found in Bermuda. Loeb made two assumptions about plant growth: that light provides all the necessary factors for plants to grow, and that the mass of plant material increases in proportion to the amount of chlorophyll. In addition, to make regeneration possible, he assumed that the amount of chlorophyll available remains constant. This was important to make clear that the conditions for growth persist through the plant’s life and not just at the beginning. These assumptions led Loeb (1924) to what he called the “mass relation,” or law of regeneration, according to which “the mass of shoots and roots regenerated varies in proportion with the mass of the leaf or stem where the regeneration occurs.” (p. 8) For Loeb, this mass relation explained how regeneration occurs, and internal factors related to polarity and the way the organism responds to environmental conditions through various tropisms explain the patterns of where regeneration occurs within the leaves.

Charles Manning Child was also intrigued by polarities within the organism, and with what he came to interpret as metabolic gradients. Child retained an emphasis on the internal organization of organisms as laying out where and how they respond to injury and repair. And while Morgan and Loeb worked for many years at the MBL, Child visited occasionally but worked at the University of Chicago, where Loeb was based.

Child did not discuss regeneration by name, but he was clearly fascinated by the ways organisms respond to injury and change as systems throughout their life cycles as individual organisms. As he put it, “The reconstitution of pieces into new individuals is fundamentally the same process as embryonic development, and the same relation of dominance and subordination exists in both” (Child, 1915a, p. 125). Child (1915b) published the relatively short *Individuality in Organisms* that followed his much longer *Senescence and Rejuvenescence* from the same year.

Unlike Morgan and Loeb, Child focused on cells and their organization within the whole organism. He felt that none of the existing hypotheses about development or recovery from injury explained the phenomena. There must be, he felt, regions within each organism with higher or lower rates of metabolism to explain change, with transmissions of “excitations” from one area to another that set up metabolic gradients. Injury or poisons or other perturbations activate transmissions across these “susceptibility gradients.”

Morgan had studied the flatworm planarians, and so had Loeb. Child did as well. Cut off their heads or tails and watch which parts grow back, Child said. This led him to conclude that “axial gradients in the dynamic processes are characteristic features of organisms” and “that a definite relation exists in each individual between the direction of the gradient of any axis and the physiological and structural order which arises along that axis” (Child, 1915a, p. 87).

Tensions, tropisms and mass relations, and metabolic gradients: all had their role in explaining what early 20th century researchers saw in regenerating organisms. Morgan, Loeb, and Child all studied a diversity of organisms, embraced materialism, and eschewed reductionist tendencies to place the causes of regeneration within very specific parts of the organism. They understood their organisms as complex living systems, and regeneration as a systems-level process that required generalized explanations applicable to more than a single species. Their combined work on regeneration also had limitations; while they drew on extensive experiments and observations, they did not have the tools to give their hypotheses more specific mechanisms in order to connect their abstract systems-thinking with fine-grained details to make them tractable for controlling regeneration.

### Toward Reductionism: Regeneration in Model Organisms

Following Morgan, Loeb, and Child, abstract ideas of tensions, mass relations, and gradients gave way to concrete observations of cell signaling and molecular genetics as investigators made use of new tools. From the mid-20th century, with a few notable exceptions, we see an inward focus, initially toward the mechanics of particular regenerating parts and cells, and later through the gene expression responsible for regeneration. This push inward has produced a wealth of information about causes of regeneration within individual model organisms but has also come with a cost. The often myopic, reductionistic attention to inner workings of specific individual parts of particular organisms has left the generalizability of regeneration explanations behind. Let’s briefly explore what this means.

By the 1950s, developmental biologists focused on the “regeneration mass,” or blastema, as a mass of undifferentiated cells that can undergo differentiation to repair damage after injury. Electron microscopy helped make this mass of cells more visible, and soon illuminated their active role during regeneration of limb muscle, for example. Further studies showed that the mass required a critical number of active nerve cells to induce regeneration (Singer, 1954; Hay, 1959).

The observable blastema also took on a theoretical role to guide explanation. In his various editions of *Developmental Biology*, Scott F. Gilbert points to the way in which the blastema came to be seen as the “progress zone” for developing limbs in particular. It provided the tangible locus for action that Morgan, Loeb, and Child had all sought (Gilbert, 2000). Yet increasing attention on cell signaling, genetic triggering, and cell differentiation of the blastema put the focus of regeneration studies on the localized, internal mechanics rather than on the whole organism.

As scientists worked on sorting out the mechanisms involved in blastema formation, a handful of biologists carried on searching for more theory-based explanations of regeneration sought by Morgan, Loeb, and Child. Most notable in this vein is Lewis Wolpert, whose work included developing a robust understanding of how positional information within cellular systems can define spatial patterns of cellular differentiation (Wolpert, 1969). Others, like French et al. (1976) expanded on Wolpert’s positional information concept of regeneration and development. While these investigators carried on the spirit of Morgan, Loeb, and Child in terms of developing explanations of regeneration that extended across individual parts and across species, their search for generalized models was the exception at the time, not the rule.

The 1980s brought flurries of activity exploring a diversity of organisms using many different methods. In the 1990s, the explosion of interest in stem cell biology, as Gilbert noted, gave the discussions new focus (Gilbert, 2000). Do organisms that regenerate easily have more stem cells, or more active stem cells, than the rest of us? Is the blastema made up of stem cells, and with what capacities? Can we finally answer Morgan’s question: whether new cells arise to take the role of damaged cells, or whether existing cells become transformed into those roles? By 1999, as Susan Bryant put it: “In the last decade, we have witnessed spectacular advances in our understanding of development in genetically tractable model systems. Given the remarkable conservation of large parts of developmental pathways, the impact of this progress reaches far beyond the organisms and systems in which they have so far been described.” Furthermore, it was time to move from simple to more complex systems for “Above all, regeneration is a problem whose time has come, because it alone has the potential to play a key role in the treatment of any or all of these complex problems, and more, but only if we understand how to induce and control it” (Bryant, 1999, p. 363).

Control: Jacques Loeb would have been enthusiastic. So would those eager to harness stem cells for medical use. Yet questions still largely focus on the nature of cells, their interactions, and the genetic pathways responsible in individual organisms and not more generally. Gradually, researchers have made enormous progress in sorting out which genes need to be expressed, in a variety of organisms, how many and which nerves can activate the blastema, how stem cells can act as sources for new cells, and other factors that make up the complexities of regeneration. These crucial components of the regeneration puzzle have been painstakingly wrought and detailed for a wide variety of organisms, giving us the kind of information that Morgan, Loeb, and Child lacked. They have also shown that there are shared suites of regenerative mechanisms involved across many species. And yet, for all of our attention to these cellular and molecular details of regeneration over the past 70+ years, the ability to harness this information to control our own regenerative abilities is underwhelming. The focus on wrestling these details from individual organisms has come at the expense of the generalizability that Morgan, Loeb, and Child embraced.

## Discussion: Lessons From History for the Future

History is often invoked as a means to avoid repeating the past, but history can also help us shape the future. What, then, can we learn from our historical quickstep? We saw how Morgan, Loeb, and Child eschewed reductionism in order to produce generalizable explanations for regeneration that were too abstract to be tractable because they lacked the tools to fill in details and refine explanations. We also saw how the past 70+ years of research on regeneration has sought to fill in those details by reducing organisms to cells and genes, and in so doing has stumbled when it has come to producing generalizable explanations. History has shown us two extremes in the arc of explaining regeneration, and now is the time to bring the two together.

One approach is to embrace regeneration as Morgan, Loeb, and Child did: as a process that occurs within complex living systems. Each organism we investigate in the laboratory is a living system, a group of parts that interact in a coordinated fashion. Types of parts could be groups of cells or molecules within a regenerating limb, but they need not be; parts and interactions can be defined at any scale, from the whole organism down to the formation and regulation of a blastema or even an individual cell. During regeneration, parts of the system interact with each other in definable ways such that some cells may initiate regeneration, activating others to proliferate, while other cells may regulate how those proliferating cells form into replacement tissues. The process of regeneration undoubtedly involves at least some different molecules, genes, and cells across axolotls, Hydra, and mice, and yet thinking about how parts and their relationships are conserved or different across these living systems is likely to yield a more generalizable understanding of how regeneration works.

What we have now is a collection of studies of many different organisms, each an organized individual system. But funding mechanisms and the structures of science tend to keep the different studies apart and make it harder to seek a shared model for all regenerating systems. We can surely learn about nerve regeneration, for example, by looking at stem cells in cancer, or germline regeneration, or limb regeneration in different organisms. We may be much closer to modeling regenerative processes overall, yet it will take work. We need ways to move past persistent pressures to specialize on one or another organism, without comparison. Ideally, efforts like this special issue can help move toward explaining regeneration in all systems.

## Data Availability Statement

The original contributions presented in the study are included in the article/supplementary material, further inquiries can be directed to the corresponding author/s.

## Author Contributions

JM provided the historical substance of this article. KM provided the framing. Both authors contributed to the article and approved the submitted version.

## Conflict of Interest

The authors declare that the research was conducted in the absence of any commercial or financial relationships that could be construed as a potential conflict of interest.

## Publisher’s Note

All claims expressed in this article are solely those of the authors and do not necessarily represent those of their affiliated organizations, or those of the publisher, the editors and the reviewers. Any product that may be evaluated in this article, or claim that may be made by its manufacturer, is not guaranteed or endorsed by the publisher.
